# A Comprehensive Review on Deep Eutectic Solvents and Its Use to Extract Bioactive Compounds of Pharmaceutical Interest

**DOI:** 10.3390/ph17010124

**Published:** 2024-01-18

**Authors:** Cátia Ferreira, Mafalda Sarraguça

**Affiliations:** LAQV, REQUIMTE, Department of Chemical Sciences, Laboratory of Applied Chemistry, Faculty of Pharmacy, Porto University, Rua de Jorge Viterbo Ferreira, 228, 4050-313 Porto, Portugal; catia2692000@gmail.com

**Keywords:** green chemistry, sustainability, neoteric solvents, deep eutectic solvents, bioactive compounds, extraction techniques, natural products, pharmaceutical industry

## Abstract

The extraction of bioactive compounds of pharmaceutical interest from natural sources has been significantly explored in recent decades. However, the extraction techniques used were not very efficient in terms of time and energy consumption; additionally, the solvents used for the extraction were harmful for the environment. To improve the environmental impact of the extractions and at the same time increase the extraction yields, several new extraction techniques were developed. Among the most used ones are ultrasound-assisted extraction and microwave-assisted extraction. These extraction techniques increased the yield and selectivity of the extraction in a smaller amount of time with a decrease in energy consumption. Nevertheless, a high volume of organic solvents was still used for the extraction, causing a subsequent environmental problem. Neoteric solvents appeared as green alternatives to organic solvents. Among the neoteric solvents, deep eutectic solvents were evidenced to be one of the best alternatives to organic solvents due to their intrinsic characteristics. These solvents are considered green solvents because they are made up of natural compounds such as sugars, amino acids, and carboxylic acids having low toxicity and high degradability. In addition, they are simple to prepare, with an atomic economy of 100%, with attractive physicochemical properties. Furthermore, the huge number of compounds that can be used to synthesize these solvents make them very useful in the extraction of bioactive compounds since they can be tailored to be selective towards a specific component or class of components. The main aim of this paper is to give a comprehensive review which describes the main properties, characteristics, and production methods of deep eutectic solvents as well as its application to extract from natural sources bioactive compounds with pharmaceutical interest. Additionally, an overview of the more recent and sustainable extraction techniques is also given.

## 1. Introduction

Everything around us is chemistry—from the atoms that comprise us, to the food we eat and the clothes we wear! Chemistry extends to all industries, whether in the pharmaceutical, textile, cosmetic, food, or agricultural areas. Thus, chemistry is one of the areas of science responsible for the development of society and the quality of life as we know it today. However, when misused, chemistry also has consequences, namely at the environmental level and for human health. Chemists are primarily responsible for the use and development of new materials and, consequently, the production of hazardous substances for the environment and humans. Thus, chemists have an increased responsibility in achieving sustainable development [1,2,3].

In the 1990s, with a view to combat all these problems, a new branch of chemistry emerged: green chemistry. The IUPAC (International Union of Pure and Applied Chemistry) defines green chemistry as “the invention, development and application of chemical products and processes to reduce or eliminate the use and formation of substances dangerous” [2]. This concept is based on 12 principles, which were introduced in 1998 by Paul Anastas and John Warner [4].

With the 12 principles of green chemistry in mind, industry in general, and the pharmaceutical industry in particular, must change their methods of production to aim towards a more sustainable product. One of the main sources of pollution of the pharmaceutical and chemical industries is the solvents that are used. Solvents are chemical substances with variable constitution from natural or synthetic sources, which are commonly used to dissolve, dilute, or disperse other compounds [5]. Solvents determine the solubility of their solutes, which is a property on which several processes are based, namely, extractions, separations, purifications, and concentration of substances. Solvents, therefore, play a crucial role in each of these processes, and are used abundantly [6].

Solvents are fundamental elements in extractions of bioactive substances from natural products, whether as dissolution reagents or simply to maintain proper molecular interactions in transformations [6]. Among the numerous solvents that can be used as extraction solvents for natural bioactive compounds, n-hexane, petroleum ether, diethyl ether, ethyl acetate, chloroform, dichloromethane, acetone, n-butanol, ethanol, methanol, and water are the most used ones. Polar solvents such as water, ethanol and methanol are commonly used as extraction solvents for polar compounds, while non-polar solvents such as n-hexane and petroleum ether are used as extraction solvents for non-polar compounds. The remaining solvents allow the extraction of bioactive compounds of intermediate polarity [7]. However, except for water, these solvents are harmful for the environment and to community health [8].

But what would be an ideal solvent?

The ideal solvent would be a fully sustainable compound, an innocuous substance without any type of negative impact, both for the environment and for humans, that simultaneously favoured the process conditions, making them less complex. To obtain it, a renewable raw material would be necessary, and the process of obtaining it would have to not generate waste or emit pollutants. Subsequently, its preparation process would have to be efficient both in economic terms and in energy terms. The application of the ideal solvent would be versatile and would contribute to the superior performance of the process. In this way, the use of an ideal solvent in a chemical process would facilitate a reduction in the amount used and allow easy elimination through recycling. The ideal solvent should be completely biodegradable, as if it had never existed. Unfortunately, we are still far from using ideal solvents [9,10,11].

Water is undoubtedly the solvent that comes closest to the ideal solvent. It is a nontoxic, non-inflammable low-cost substance. But water has also some disadvantages, such as it does not dissolve nonpolar compounds due to the high dielectric constant (at room temperature and normal pressure), and it has a high boiling point which entails a high energetic cost for its removal. Additionally, the scarcity of water in some regions of the world and the possible contamination of the effluents due to difficulties during the separation processes are also problems associated with the use of water as a solvent [12,13,14].

The simplest solution to the solvent use problem would be to develop methods that do not require solvents, such as mechanochemical methods [15,16]; however, this is not always feasible, and therefore, these compounds should be avoided or replaced by innocuous solvents for the same purposes [1,17].

## 2. Deep Eutectic Solvents

Neoteric solvents emerged as an alternative to organic solvents and are commonly called green solvents, since they have low toxicity, are biodegradable, are made with accessible and low-cost materials, and are quite easy to produce [10,11]. This class of solvents follow some of the 12 principles of green chemistry, as they come from a natural origin have low toxicity, are biodegradable, and minimize waste as much as possible. Among them are supercritical fluids, thermomorphic solvents, switchable solvents, liquid polymers, solvents derived from biomass, fluorinated solvents, ionic liquids (ILs), and deep eutectic solvents (DESs) [11,14,18].

In this review paper we will focus on DESs; however, several reviews can be found on neoteric solvents and their applications [10,14,19,20,21,22,23].

The first deep eutectic solvent (DES) was a mixture of choline chloride (T°m = 302 °C) and urea (T°m = 133 °C) in a 1:2 ratio, which led to the formation of a transparent liquid with a melting point of 12 °C, much lower than that of its individual constituents, and which exhibited quite different properties [24].

**Figure 1:**
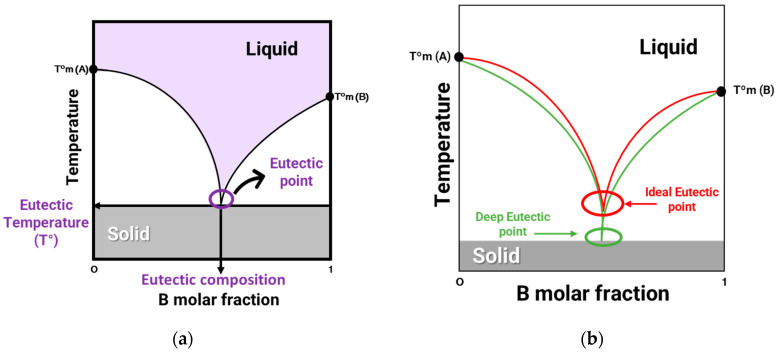
(**a**) Solid–liquid phase diagram of a binary mixture of compounds A and B. (**b**) Phase diagram of typical ideal eutectic mixture (red) and a deep eutectic solvent (green).

The concept of eutectic mixture is based on the word eutectic, originating from the ancient Greek word *eutēktos*, which means “easily melted” and is interpreted as the decrease in the melting point after the combination of its constituents [23]. A eutectic mixture is defined as a mixture of two or more components that results in a significant decrease in the melting point compared to the initial pure compounds [11]. The eutectic system can be represented through a phase diagram of temperature as a function of the composition of the eutectic mixture (Figure 1a). As the two compounds, A and B, mix, their respective melting temperatures (T°m (A) and T°m (B)) decrease until they reach a minimum point, called the eutectic temperature of the mixture. The eutectic point represents the composition and minimum melting temperature at which the melting curves of these two constituents intersect. A DES (Figure 1b) is a non-ideal eutectic mixture in which an abrupt depression of the eutectic point occurs, hence the term deep [23,25]. To define what is an ideal eutectic mixture, theoretical calculation must be performed to know the theoretical eutectic point (red curve in Figure 1b). When two compounds are mixed and become liquid in the eutectic point, it is inferred that we are in the presence of a non-ideal eutectic mixture, hence a deep eutectic solvent (green curve in Figure 1b) [26].

DESs are defined as a mixture of two or more pure compounds, which, when combined in an appropriate ratio, give rise to a eutectic mixture that deviates from the ideal thermodynamic behaviour. This deviation is due to strong interactions between the initial components that act as hydrogen bond donors (HBDs) and hydrogen bond acceptors (HBAs). The HBDs and HBAs interact in the DESs to form a dense network of molecules that give them remarkably interesting physical and chemical properties. These properties include a low melting point, low volatility, high thermal stability, and a high solubilizing power of a wide range of compounds, namely those poorly soluble in water [17,25]. DESs are also customizable solvents, meaning that their physical and chemical properties can be adjusted by changing their constituents. Consequently, it is very easy to obtain a large variety of DESs with different properties and applications, always with a maximum efficiency [23,27].

**Figure 2:**
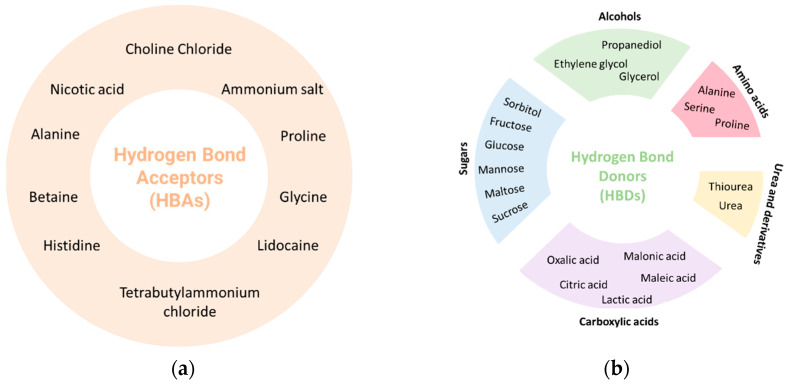
Typical (**a**) hydrogen bond acceptors (HBAs) and (**b**) hydrogen bond donors (HBDs) used in the preparations of DESs.

The most used HBA is choline chloride due to its low cost and biodegradability [28]. Choline chloride is the most economical synthetic way to obtain choline and can be produced on a large scale, it can be extracted from biomass, or it can be synthesized from fossil reserves [29]. However, other compounds are also used as HBAs, namely betaine, alanine, glycine, lidocaine, among others. Regarding HBDs, compounds such as thiourea, urea, citric acid, malic acid, tartaric acid, glycerol, menthol, xylitol, and oxalic acid are often used (Figure 2) [25,30]. Water can also be present in its composition and can play various roles, such as HBA, hydrogen bond donor (HBD), or simply as a dilution solvent [25].

DESs have several advantages when compared with other neoteric solvents, as for example ILs. DESs are extremely easy to prepare with a high purity and a 100% atom economy without forming any by-product [11,31]. DESs do not require any purification step or waste disposal, and they have a low toxicity, thus reducing adverse environmental and human effects [11].

The main disadvantages of DESs are their high density and viscosity [27,32], which reduce their fluidity, making it difficult to transfer solvents and masses, especially in continuous operations, and in dissolutions [6]. This obstacle can be overcome by applying external physical forces such as microwaves and high temperatures or via the addition of water [27,32]. Furthermore, low vapor pressures contribute to the low volatility of DESs, which makes it difficult to separate them when needed. This especially important in their use as extraction solvents.

The applications and potential applications of deep eutectic solvents are immense, highlighting their use as solvents [33], as extraction solvents [34,35,36,37,38,39,40,41,42], as electrolytes for lithium batteries [43], in nanotechnology [44], in gas capture [45], for the synthesis of new materials [46,47,48], in chromatography [38,49], for electrochemical analysis [50], in biomedical applications [51], for removing excess glycerol in biodiesel [52], and to mediate organic reactions [53]. More recently, deep eutectic solvents have been applied in the pharmaceutical industry with the aim of improving the bioavailability of drugs [54]. This is achieved by increasing the active pharmaceutical ingredient (API) solubility [55,56,57], permeability [58,59], and stability [60,61], as well as through the controlled release of drugs [62,63].

In this review, we will focus on the applications of DESs for the extraction of bioactive compounds with pharmacological interest.

### 2.1. Deep Eutectic Solvents Classification

DESs can be classified as hydrophobic or hydrophilic according to their solubility in water [11]. The majority of the DESs are hydrophilic due to the extensive network of hydrogen bonds [23,64]. Hydrophobic DESs are defined as insoluble or very poorly soluble substances in water, composed of two or more compounds insoluble in water [23,65]. Hydrophobic DESs have been successfully applied in several areas, namely for water purification [66], in the preparation of new materials such as magnetic gels, in nanoparticles consisting of carbon nanotubes and graphene for the removal of organic micropollutants and metallic ions from water [67,68], in the capture of carbon dioxide [69], in electrolyte medium for solar cells [70], and for the extraction of bioactive compounds [71,72].

DESs are usually classified according to the type of compounds used in their preparation and are subdivided into four subclasses: natural deep eutectic solvents (NADESs), therapeutic deep eutectic solvents (THEDESs), polymeric deep eutectic solvents (PDESs) and poly-quasi eutectic solvents (PQDESs) [23].

NADESs were discovered in 2011 when trying to elucidate the solubility of intracellular compounds, which were insoluble in water and lipids [23]. NADESs contain in their composition cellular metabolites such as amino acids, alcohols, sugars, and organic acids [28,32,73]. In addition, water can also be part of its composition, forming a ternary system [25]. In nature, we can easily find this type DESs in different cells and organisms. For example, nectar is nothing more than a mixture of sugars that are solid at room temperature when separated, but liquid when combined. Another example is honey, with such interesting and unique properties that are not yet fully understood, but with tested medical applications [32,74,75,76]. NADESs play a key role in cellular metabolism and in many biological processes such as resistance to drought, germination, and dehydration. In addition, all living organisms have a process called organ cryopreservation, which is a defence mechanism to withstand extreme conditions, such as temperature variations between winter and summer. NADESs act as cryoprotective agents for the simple fact that membranes, enzymes, and metabolites remain stable with the addition of this type of eutectic mixtures [23,28]. In terms of applications, NADESs have been used in biocatalysts processes [77,78], in the extraction of compounds [79,80], in the pre-treatment of biomass [81,82], in electrochemistry for the detection of bioactive materials [83], for drug solubilization [84], for permeation enhancement [85], and as extraction solvents [17,30].

THEDESs emerged as one of the strategies to promote the increased solubility, permeability, and, consequently, bioavailability of drugs [86,87]. THEDESs are a class of DESs that use at least one active pharmaceutical ingredient (API) as one of its components [62,88]. These solvents have raised a lot of interest and THEDESs are currently being studied, namely for increasing the solubility of drugs in aqueous solutions or increasing their permeability in different biological barriers such as the skin or intestinal wall, among others [55,85,89,90].

Another class of DESs are polymeric, so named because a portion of DES is polymerizable [23,91,92]. The polymer, when completely converted, can be used in various applications, namely in nanotechnology [93], electrochromatography [94], and gas capture [91]. In 2017, a new class of DESs was proposed: the quasi-polymeric deep eutectic solvents [91].

### 2.2. Deep Eutectic Solvents Synthesis

**Figure 3:**
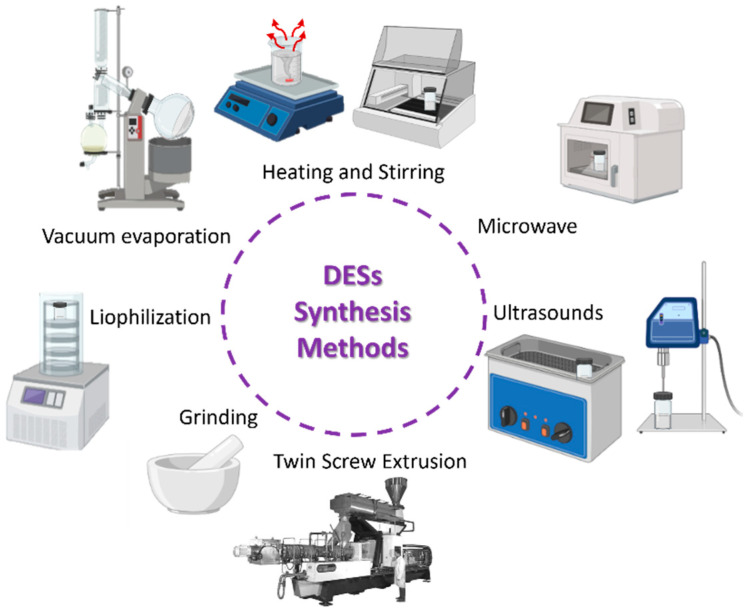
Deep eutectic solvent synthesis methods.

DESs can be synthesized in various ways depending on the equipment available (Figure 3). Independently of the type of equipment that is used, the synthesis involves the mixture of two (or more) components normally without the need of any solvent, and then energy is provided to the system for a certain amount of time in the form of a temperature increase (heating and stirring), irradiation (microwave and ultrasound), mechanical forces (grinding), or a combination between temperature and mechanical forces (twin screw extrusion). There are also methods in which the initial components are dissolved in a solvent (normally water) and then heated in a vacuum to evaporate the solvent (vacuum evaporation), or frozen and lyophilized (lyophilization) [23,27]. Another method that seems interesting in terms of sustainability is the use of concentrated solar radiation [95].

The temperature is important, and it should be carefully chosen due to the possibility of degradation of the initial compounds [23,25,96].

The time needed for the DESs to be synthesized may vary from minutes to hours depending on the method of preparation and on the initial components and their ratio.

### 2.3. Deep Eutectic Solvents Properties

Deep eutectic solvents have a set of properties that make them quite useful as extraction solvents. One of the features of DESs is the possibility of being used as extraction solvents for a wide range of solutes [10]. The main characteristic that makes them good extraction solvents is their solvation capacity, that is, the fact that they can accept and transfer protons and electrons, establishing hydrogen bonds with the compounds and retrieving them from their matrix [97]. DESs are known for their enormous capacity to dissolve very poorly soluble metabolites in water. They are also able to dissolve natural products such as rutin, paclitaxel, gingilido b and quercetin, starch, deoxyribonucleic acid (DNA), and high-molecular-weight proteins [30]. Dai et al. [98] verified that small molecules, such as rutin, paclitaxel, gingilido b, increased the solubility values in DESs when compared with water. It was found that, for example, rutin is 50–100 times more soluble in DESs than in water. DESs are also capable of stabilizing natural products. Natural pigments such as carthamine are more stable to light, elevated temperatures, and storage time in various DESs with sugars than in water or a 40% ethanol solution [99]. The same stabilizing effect was later observed in anthocyanins [100]. Recently, the effects of DESs in the stability of phlorotannins extracted from F. vesiculosus was studied for 360 days and compared with their stability and ethanol. It was found that the DESs enabled greater stability than ethanol [101].

Polarity expresses the strength of a solvent; that is, it determines its solvation power and is an important characteristic for a solvent [25,28,102]. The polarity of a DES can be adjusted by changing its constituents, making it more polar or apolar accordingly to necessity, improving the selectivity of the solvent towards a particular bioactive component or class of components. A relative polarity scale could be established, but there are few publications about the polarity of DESs. Among the most used scales is the Dimroth and Reichardt scale; however, these scales are not universal and depend on probes. This means that the polarity parameters obtained by different probes cannot be compared [23,25,103]. Variations in the polarity of DESs depend on the compositions of their individual constituents and are believed to be related to the molecular structure of the HBD [102,103]. As a rule, polarity increases with increasing intermolecular attractions. Omar et al. [103] found that for the same DES choline chloride/glycerol in different molar ratios of 1:1, 1:2, and 1:3, the polarity values were of 58.49 kcal/mol, 58.00 kcal/mol, and 57.96 kcal/mol, respectively.

The thermal stability of DESs is an important property because it limits the maximum operating temperature at which DESs can be useful. Between the temperature of glass transition (Tg) and the decomposition temperature, the DES maintains its liquid state and the properties that arise from that condition [104]. Delgado-Mellado et al. [104] studied the thermal stability of eight different choline chloride-based DESs and found out that the volatility of the HBDs was the main contributor to the decomposition of the DESs. The authors also emphasized the importance of establishing the real range of operational temperatures for DESs to be able to use them at the industrial level. All DESs are normally glass formers with a Tg below 0 °C; however, this property can be modified with the inclusion of water in the DESs structure due to the plasticizing effect of water. Craveiro et al. showed that an increase of 5 wt% of water in a chlorine chloride/xylitol (2:1) DES decreased the T_g_ by 4 °C [105]. The presence of water (added or absorbed during preparation) can also influence the thermal stability of the DESs if the water is lost upon heating. This can be observed in thermogravimetric analysis with mass lost around 100 °C due to water evaporation [106].

Water plays a significant role in the physicochemical properties of DESs, and the incorporation of water into DESs either by adding it intentionally or by its absorption from the ambient air is inevitable; therefore, several authors have studied the role of water in DESs. Water can be an HBD or HBA and, in this way, can be a part of the structure of the DES, or it can play the role of the solvent used to decrease the density and viscosity of the DESs. Edler and al. [107] were the among the first to study the effect of water in DESs by studying a series of choline chloride/urea/water DESs by neutron total scattering and empirical potential structure refinement. They found out that until 42 wt% of water the DESs nanostructure is maintained due to the solvophobic sequestration of water into nanostructures domains around cholinium cations. At 51 wt% of water, this segregation is disrupted, and DES–water interactions are dominant, and above this level of water, the mixture was described as an aqueous solution of DESs components. The role of water as an additional HBD was shown by López-Salas et al. [108]. They studied the role of water in a ternary DESs system of resorcinol, urea, and choline chloride by ^1^H NMR and Brillouin spectroscopy. They realized that the tetrahedral structure of water was distorted as a consequence of its incorporation as an additional HBD or HBA. This fact was confirmed by DSC showing the formation of a new eutectic solvent with a lower melting point when water was incorporated.

Water can also be incorporated into hydrophobic DESs, altering their properties. Kivelä et al. [109] studied the low water absorption by a 1:2 molar ratio of tetrabutyl ammonium chloride and decanoic acid and found out that even extremely low water content causes nanoscale phase segregation, reducing viscosity and fragility, increasing self-diffusion coefficients and conductivity, and enhancing local dynamics. Water interferes with the hydrogen bonding network by solvating the carboxylic acid group.

The existing studies show that physicochemical properties of DESs can be tailored by adding water in a controlled way [110]. The incorporation of water in the DESs can decrease viscosity by enhancing mass transfer from solid samples to the solution and increasing the extraction efficiency, but it also increases the polarity of the DESs making it more suitable for extracting more polar components [111]. However, if too much water is incorporated into the DES, the hydrogen bond network between the DES components can be disrupted ending up with an aqueous solution of the DES components [110].

The toxicity of any compound depends on its ability to cross or interact with biological membranes and is affected if its structure is disrupted [112]. DESs are considered green solvents, presenting a low toxicity, which comes from the use of initial constituents of natural or little toxic origin. However, studies of the toxicity, cytotoxicity and ecotoxicity of DESs and respective aqueous mixtures are still too rare for them to be classified as safe [28,113,114]. Initial studies showed that DESs were biodegradable and non-toxic [115]. However, some DESs proved to be more toxic than their initial constituents [116].

Toxicity and cytoxicity studies made with DESs composed by different HBAs and HBDs in Gram-positive and Gram-negative bacteria and shrimp larva gave diverse results depending on the DESs. Some DESs were shown to be toxic for some of the bacteria used, and their toxicity was associated with the pH and with the charge delocalization between the HBA and HBD. The DESs also showed higher cytoxicity when compared to the initial components. The authors concluded that the lack of oxygen and their high viscosity may be the reason for this behaviour [116]. Fish cells and a human cell line were used to study the toxicity of three choline chloride DESs. One of the DESs showed a moderate toxicity due to the formation of calcium ions in addition with a pH decrease when the DES was added to the culture medium [115]. Lapena et al. [112] studied and evaluated the ecotoxicity of six DESs on algae, bacteria, and crustaceans. The authors concluded that the inclusion of water in the DESs can change the DES toxicity because water can be a part of the DESs or can disrupt the intermolecular forces between the DESs’ components. Sanches et al. [117] performed an ecotoxicological screening of 15 DESs using an extensive set of marine and freshwater bioassays. The main conclusion was that none of the DESs presented toxicity; however, both algal assays showed a certain degree of biostimulation, up to over a 100% growth increase in respect to controls, with 8 out of 15 compounds tested with *Raphidocelis subcapitata*. Therefore, their release into aquatic systems may represent a risk leading to ecosystem functioning impairments.

Juneidi et al. [118] evaluated the toxicological profile of ten DESs on fungi and establish that the toxicity of the acidic DESs was higher since it is known that acid compounds can cause cell membrane and protein damage. Nevertheless, the DESs showed a lower toxicity than the respective acids when used isolated. The authors consider that this decrease can be explained by a pH change during the formation of the DESs or by a synergetic effect between the two initial compounds. The acidity of DESs was also considered a problem in a study made by Passos et al. [119] which evaluated the toxicological profile of nine DESs on an enzyme. All the DESs were constituted by sugars, organic acids, and water. The variation in the sugars was found to have no relation with the toxicity of the DESs; however, the acidity of the organic acids was linked to have a direct relation with the increase in the DESs toxicity. Zhao et al. [120] studied twenty DESs that contained amines, alcohols, sugars, and organic acids as HBDs. Toxicity was evaluated for Gram-positive and Gram-negative bacteria. All DESs containing amines, alcohols, and sugars as HBDs showed no inhibition of any of the bacteria. Only the DESs constituted by the organic acids as HBDs significantly inhibited all bacteria (seven DESs out of twenty). Higher inhibition was found for Gram-negative bacteria. The authors concluded that the characteristic acidity of these compounds must be responsible for the damage caused to their outer membrane. This is extremely important, since Gram-negative bacteria have their own external membrane, which acts as a protective barrier, making them more resistant to external aggressions; therefore, these DESs are a hypothesis to combat this type of very resistant bacteria. Li et al. [121] proposed a rating scale for DES toxicity: Type 1, Type 2, and Type 3. Type 1 is the DES that has a higher toxicity then the individual constituents due to new interactions created during the formation of DES. Type 2 is the DES that has lower toxicity then the initial constituents. In this case, the properties that make the initial components toxic are modified in the DESs. Finally, Type 3 is the DESs whose toxicity is the combination of the toxicity of its constituents. The authors studied DESs with amino acids in their constitution and observed, for the first time, that DESs containing amino acids can also present toxicity.

Polar DESs are capable of co-extracting trace elements of metals during the extraction process; however, this has rarely been investigated. Shikov et al. [122] studied the ability of acid-based DESs to co-extract metallic elements from the roots of *Glycyrrhiza glabra* L. and its associated health risks. The authors found that several metals were co-extracted; however, the amount of metallic elements did not pose any health risks. According to the study, the HBA played a decisive role in the extraction of these elements. This type of toxicity should be further investigated since different types of DESs can co-extract elements that can be toxic for human health.

There are few studies that evaluate the biodegradability of DESs. All DESs biodegradability studies follow the Standard OECD No.301 D, which allows the classification of a compound as easily biodegradable or not in an aqueous aerobic medium. According to this standard, to consider a material easily biodegradable, it is necessary that the level of biodegradation is 60% on the 10th day out of 28 for respirometry methods [123]. Lapena et al. [112] studied the biodegradability of six DESs and concluded that the addition of water to the DESs affects their biodegradability, increasing or decreasing it depending on the DES. The number of hydroxyl groups was also a factor in the percentage of biodegradability of the DESs. The same conclusion was retrieved by Radošević et al. [115], who evaluated the aerobic biodegradability of three DESs with components containing different number of hydroxyl groups. The authors concluded that the higher the amount of hydroxyl groups, the higher the percentage of biodegradability.

The density of the DESs is an extremely important property due to the implication it has on their use and handling. Very high densities can cause the DESs not to flow, which can impair their processing. The density values of DESs are higher than those of their pure constituents, and as a rule, DESs have values higher than those of water, except for hydrophobic DESs [25,102,103,124,125]. DESs are usually highly viscous solvents, which can impede mass transfer and decrease the extraction efficiency [97]. Viscosity translates resistance to deformation at a given shear rate of a given fluid [11,96,126,127]. A liquid with low viscosities flows very easily, while more viscous ones flow more slowly. This is particularly important, as it will influence and determine its commercial applicability and the cost of the process [28,125,126]. The high density and viscosity of DESs can be circumvent by adding water to the DESs and/or handling them at temperatures higher than the ambient temperature.

One of the characteristics of the DESs is their low vapor pressure. This intrinsic characteristic of these compounds can be an advantage or a disadvantage depending on the application. In an extraction process, it is preferable that the DESs have a lower vapor pressure, considering that the extraction temperature is reached without the loss of extraction solvents by evaporation. However, if we intend to separate the DES from the extract a posteriori, a low vapor pressure is a disadvantage because it hinders its evaporation, unless the DESs can be incorporated in the extracts or other methods are used to separate the DESs from the extracts [6,128,129].

## 3. Bioactive Compounds

Bioactive compounds are secondary metabolites that are present in plants, fungi, microorganisms, and animals and can cause pharmacological or toxicological effects in humans and animals [35,130,131]. Phytochemicals are bioactive compounds present in plants, such as fruits, vegetables, and cereals [132]. These compounds are classified into phenolic compounds (polyphenols), terpenoids and nitrogen-containing compounds. Phenolic compounds are the largest group of phytochemicals and are present in almost all plants in the form of secondary metabolites, where they play a key role both in growth and reproduction processes, as well as in protection against pathogens and predators [128,133,134]. Phenolic compounds can in turn be divided into flavonoids (anthocyanins, flavonols, flavones, isoflavones, and flavols) and non-flavonoids (phenolic acids, stilbenes, lignins, and tannins) [135].

**Figure 4:**
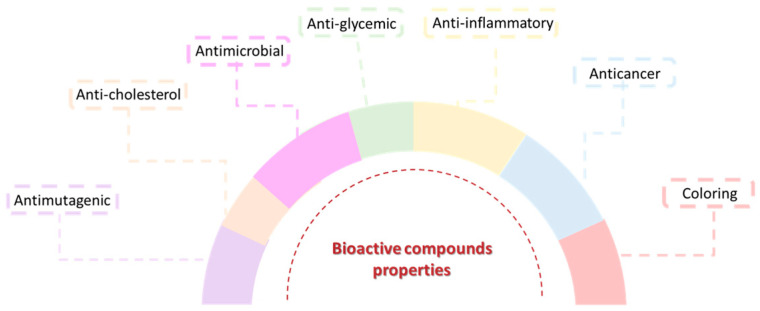
Some of the main properties of the bioactive compounds.

Bioactive compounds have been extensively investigated and their application as therapeutic agents has increased due to the important biological properties they possess, namely anti-inflammatory, antidiabetic, analgesic, anticancer, antimicrobial (antifungal and antiviral) and antioxidant activities, which makes them useful for the prevention of various diseases such as cardiovascular diseases [136], neurodegenerative diseases [87], and cancer [137]. Some of these compounds have also been shown to have anti-aging properties [138] and the ability to absorb ultraviolet radiation through the presence of chromophores in their composition, causing the entry of radiation into the skin to be blocked by these compounds, protecting it [139]. Hair growth-promoting [140] and nail damage prevention [141] properties have also been reported. These characteristics make them extremely interesting compounds for applications in the pharmaceutical industry [131,133] (Figure 4).

### 3.1. Extraction of Bioactive Compounds

**Figure 5:**
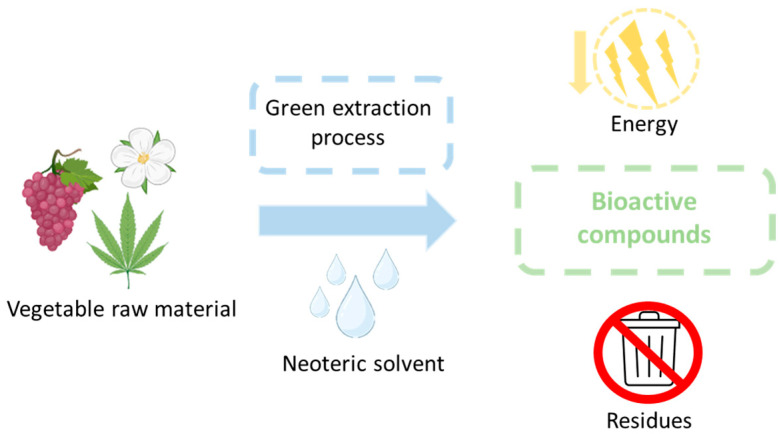
Process for obtaining bioactive compounds. Adapted from [142].

To obtain bioactive compounds, it is necessary to extract them from natural sources (Figure 5). The objective is to obtain sustainable compounds, so the entire process involving their extraction must comply with this requirement. This implies a raw material of natural and environmentally friendly origin, an environmentally friendly solvent and extraction method, and the lowest possible energy expenditure with the lowest possible waste production [142,143,144].

#### Extraction Techniques

**Figure 6:**
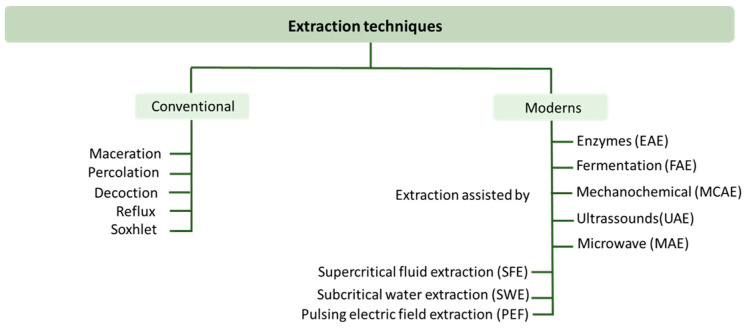
Techniques normally used for the extraction of bioactive compounds.

The extraction of compounds with a bioactive potential is the first step in the research and development of new natural products. These extraction methods must be selective, economical, reproducible, environmentally friendly, safe, and effective [145]. Natural bioactive compound extraction techniques are divided into conventional and modern techniques (Figure 6).

Maceration, percolation, decoction, reflux, and Soxhlet are some examples of conventional extraction methods and use solvents such as water or organic solvents such as methanol, ethanol, propanol, acetone, and ethyl acetate [145,146]. The non-selectivity of these conventional methods, the possibility of degradation or isomerization of the bioactive compounds due to prolonged heating, their low extraction yield, the negative environmental effects, their long extraction times, the high extraction costs associated with large amounts of organic solvents and energy costs, and the wrong methodologies for recycling the hazardous solvents that are used have made it necessary to develop other more environmentally friendly techniques [131,145,146].

The so-called modern techniques were developed with the intention to reduce the use of toxic solvents, reducing the waste and energy consumption, and at the same time increasing the extraction yield [102]. New methods were then proposed, such as enzyme-assisted extraction (EAE), fermentation-assisted extraction (FAE), mechanochemically assisted extraction (MCAE), ultrasound-assisted extraction (UAE), microwave-assisted extraction (MAE), supercritical fluid extraction (SFE), and subcritical water extraction (SWE).

Enzyme-assisted extraction (EAE) is a biological extraction method that is based on the ability of enzymes to hydrolyse plant compounds, breaking their cell wall to release the cytoplasmic content, namely bioactive compounds, in an extraction solvent. Regarding its advantages, this extraction is a specific technology, given that the enzymes used are specific, such as cellulase, α-amylase, hemicellulose, pectinase, among many others [145,147,148,149]. Most of these enzymes are derived from microbial organisms or other various sources, such as plants and animals [102]. However, and this being its biggest disadvantage, some enzymes are expensive, especially on a large scale or on an industrial scale if there is a large substrate enzyme ratio, making this method expensive [149].

In fermentation-assisted extraction (FAE), microorganisms are used for fermentation of substrates in plant materials to facilitate the selective extraction and separation of target biomolecules from their complex matrices. Only microorganisms that already exist in the material itself are used, so it has a low associated cost. However, this technique is not specific, and sometimes there is formation of by-products or hydrolysis of the target compounds [145].

The extraction of pulsed electric field (PEF) is based on the application of intermittent pulses of high voltage current during short periods of time, in the order of micro to milliseconds, in a previously placed inside a chamber, located between two electrodes. This voltage generates an electric field, and its intensity is dependent both on the distance between the electrodes and on the applied voltage. When the electric field exceeds the critical value, an electroporation phenomenon occurs. This phenomenon consists of the increased permeability of the cytoplasmic membrane to the passage of ions and macromolecules due to the repulsion between charged molecules. Plant materials are destroyed by disruption of their membrane and there is an increase in mass transfer during extraction, reducing extraction time. The main advantage of this method is the non-use of heating, which minimizes the degradation of thermolabile compounds and requires less energy. Moreover, the addition of chemicals is not necessary, which reduces the associated cost without affecting the quality of the product. This technique has some disadvantages such as an exceedingly high initial cost and a dependence on the conductivity of the environment [148].

Currently, mechanochemical methods are attracting a lot of attention due to the much higher extraction yields they present, their low cost, short extraction time, mild experimental conditions, simplicity of equipment, and ease of coupling a posteriori with various methods of analysis. Mechanochemically assisted extraction (MCAE) is an extraction method that consists of applying a mechanical force (high-speed grinding) to the tissues and cell membranes of plant materials. This force facilitates the release of intracellular molecules and consequently the extraction of target compounds from their matrix. A solvent can be used in extremely low amounts to react with the target compounds under mechanical force improving the extraction yield. The purpose of this technique is not simply to increase the contact area between the target compounds and the solvent, but also to chemically transform them into forms that are more soluble in water or in the solvent [145,150]. The main advantages of this method include the simplicity of the extraction process, the use of a lower extraction temperature that allows the extraction of thermolabile compounds and reduces the cost of the process, a shorter extraction time, the use of low amount of solvents, if needed, and an increase in the selectivity of the extraction [151,152]. This technique proves to be far superior in the fragmentation of cells and tissues, homogenization of materials. However, the method has disadvantages, namely the difficulty of extracting compounds that are hydrophobic and neutral [145].

Ultrasound-assisted extraction (UAE) is one of the most used modern extraction techniques and is a technique already used to extract bioactive compounds since 1980. This method is based on the use of low-frequency energy (20–1000 kHz) and high power (80–200 W), ultrasonic waves [102,148,153]. Upon application and penetration of ultrasonic waves into the plant matrix, cycles of compression and expansion occur in the medium that result in the phenomenon of cavitation and consequently the formation of air bubbles. The size of these bubbles varies during the expansion and compression cycle until it reaches its critical size and collapses with consequent release of energy. This causes the rupture of the cell wall of the plant material and the release of the bioactive compounds that are inside the cells [73,146,154]. As advantages, this method is quite simple since it only needs an ultrasonic bath or an ultrasonic probe, and a dispersive solvent is not necessary to increase the surface area of contact between the extraction solvent and the sample [10,102,153]. In addition, it uses low amounts of solvent, consumes little energy and the extraction times are short. The phenomenon of cavitation improves the efficiency of the extraction, as it not only accelerates the dissolution and diffusion of bioactive compounds, but also allows heat transfer. Thus, significantly higher extraction yields are obtained than with other techniques. As disadvantages, this method is not applicable to thermolabile compounds, and the noise from ultrasonic waves is annoying for the operator [147].

Microwave-assisted extraction (MAE) consists of using microwaves with frequencies in the 0.3–300 GHz range, which interact directly with the sample through dielectric heating. In this process, microwave electromagnetic radiation heats a dielectric material through a dipole rotation of the polar components present in the matrix. In this way, it leads to the degradation of plant cell tissues and induces the flow of ions, releasing the active compounds from the intracellular and cellular membrane [73,102,146]. With regard to its advantages, it is an easy-to-handle and efficient technique, with high extraction yields, short extraction times, low energy expenditure, and a lower consumption of solvents. In contrast to some other techniques, it can analyse several samples simultaneously, which leads to a low extraction time [73,145]. Its major limitations are the fact that it is not applicable to thermolabile compounds or production at an industrial level due to the associated cost and to the impurities of the extracts obtained due to the intense extraction conditions that make it possible to extract different analytes, even the undesirable ones [145,146].

When a gas undergoes compression and heating, its physical properties as a gas change and it becomes a supercritical fluid. This fluid has the solvation power of a liquid and the diffusivity of a gas at temperature and pressure below the critical point [148,149]. The supercritical extraction technique is based on the introduction of a supercritical fluid into a plant material that will extract bioactive compounds according to their solubility. Co-solvents such as water, ethanol and methanol can be added to increase the solvation power of the supercritical fluid, which may allow the extraction of polar compounds [148,149]. Supercritical carbon dioxide is the most used. It allows the extraction of lipophilic compounds and fats, without the need for a concentration step [153]. In terms of advantages, the method uses green solvents without the need to use organic solvents which makes it environmentally friendly, the process can be automated, it is selective, it prevents sample oxidation, the extracted compounds are very stable, and it uses low temperatures. Furthermore, the low viscosities and high diffusivities of these fluids increase mass transfer, reducing extraction time. As disadvantages, the technique has a high associated cost, the equipment used is complex, consumes a high amount of energy, and has low selectivity for polar compounds [148,149].

Subcritical water extraction (SWE) consists of extracting less polar compounds using water as the extraction solvent. Subcritical water is kept in liquid state under high pressure (10–60 bar) and temperature (100–374 °C). At 25 °C, the dielectric constant of water is 80. If the temperature is increased to, for example, 250 °C and the pressure to 25 bar, the dielectric constant drops dramatically to 25, lying between that of methanol and ethanol, at 25 °C. In this way, at these temperatures and pressures, water can extract compounds of medium to low polarity [148,155,156,157]. The advantages of this extraction method are its simplicity and the fact that it only uses water as a solvent, which makes the extraction technique green and with a lower associated cost. Polar, moderately polar, and non-polar compounds can be extracted separately using this technique, which is a great advantage, for example, compared to supercritical extraction. In addition, the technique has a short extraction time, high efficiency, and enables a continuous process. However, it has disadvantages, namely the difficulty in separating the bioactive compounds from the extracts, the thermal degradation and oxidative damage that can occur when using high temperatures, and the difficulty in cleaning the equipment [148,157].

## 4. Deep Eutectic Solvents for the Extraction of Bioactive Compounds

**Figure 7:**
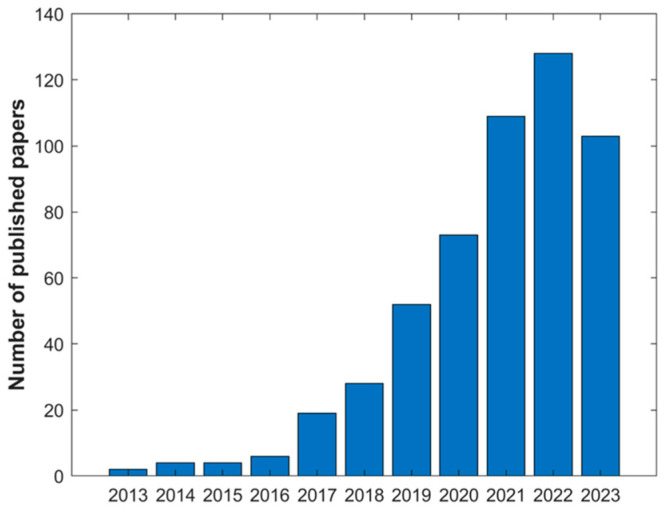
Number of papers in Web of Knowledge on 21 November 2023 with the search “Deep Eutectic Solvents Bioactive Compounds Extraction”.

The use of DESs for bioactive extraction gained a lot of interest by the scientific community in the last 10 years. A rapid search in the Web of Knowledge platform with the keywords “Deep Eutectic Solvents Bioactive Compounds Extraction” shows a rapid increase in the number of papers published in this last decade (Figure 7).

The increase used of these solvents is due to their unique chemical properties such as low melting point, low vapor pressure, chemical and thermal stability, polarity, and miscibility solubility. Moreover, they present a 100% atom economy, low environmental impact due to their low cost, and simplicity to produce using natural and environmentally friendly substances [158]. Polarity is a key property to solubilizing compounds in an extraction process. The HBAs and HBDs used to for the DESs play a significant role in the final polarity, as well as the addition of water [98,159]. For example, Craveiro et al. showed that DESs composed of choline chloride and organic acids were more polar than the ones with sugars [105]. Depending on the target compound, the DESs can be more hydrophobic or hydrophilic. Most of them are hydrophilic, but the use of hydrophobic DESs in extractions is increasing [160]. Hydrophobic DESs based on choline bromide with different chain lengths were synthesized to extract phytochemicals from *Cannabis sativa*. The hydrophobic and hydrophilic features of the DESs facilitated the interactions with polar and non-polar compounds present in the plant increasing the extraction efficiency [161].

With the keywords referred in the beginning of this section and including only the papers published in 2023 it can be seen that many applications were developed to extract bioactive compounds from natural sources with pharmaceutical interest using DESs (Table 1).

From the components used to synthesize the DESs, choline chloride as the HBA was by far the most used one; however, caution must be taken when using this compound since it is forbidden for cosmetic applications [162,163].

Most of the authors found that the extractions with DESs, independently of the extraction method, have high yields when compared with the use of traditional solvents (methanol, ethanol, acetone, etc.) with advantages of being used at milder temperatures without flammable solvents and lower extraction times [164,165]. Liu et al. [166] screen twenty-seven DESs combined with UAE to retrieve scutellarin from *Erigerontis Herba*, an herb used in Chinese medicine. From the DESs screened, twenty-five had a higher extraction yield than the conventional solvents (methanol and 75% ethanol solution). Vinci et al. [167] used DESs to extract antioxidant compounds from dark chocolate. The DES of betaine and choline chloride showed a 35% higher extraction yield than traditional solvents.

Evidently, there are several factors that influence the extraction yield and components extracted. Some of the most influential are the biomass-to-solvent ratio, the time and temperature of the extraction, the type and ratio of HBA and HBD, the viscosity and density of the DES, water content, and pH [165,167,168]. Chagnoleau et al. [162] screened fifteen DESs for extracting antioxidants from out-of-calibre kiwifruits. The authors studied the influence of water on the extraction and demonstrated that up to 40% of water could be added to the system without compromising the antioxidant activity of the extracts. The number and type of hydrogen bonds present in the DESs have a profound impact, not only in the extraction yield but also in the nature of bioactive compound to be extracted. Theoretical studies demonstrated that hydrogen and π-bonds were the main factors affecting the extraction of hesperidin from orange peel using a DES of triethanolamine/4-methoxyphenol (1:1) with a water content of 35% [169].

One of the advantages associated with DESs is their capacity to be tailor-made solvents, i.e., by varying the HBA and HBD different bioactive compounds can be extracted [170]. This selectivity of the DESs can be explained by different chemical interactions with the target compounds [165]. Ojeda et al. [171] used mango by-product to extract bioactive compounds using DESs. To understand the relation between viscosity and the hydrogen bonds in the DESs, the authors performed a theoretical study and found out that the hydrogen bonds have a determining effect on the viscosity of the viscosity of the DES and in the specificity of the compounds extracted. The selectivity of the DESs in extractions was showed by Santos-Martín et al. [172], who used two DESs, one composed of lactate, sodium acetate and water, and a second composed of choline chloride and oxalic acid, to extract phenolic compounds from blueberry leaves using UAE. The authors conclude that not only did the DESs display a superior performance for the recovery of phenolic compounds when compared to traditional organic solvents, but also the lactic-based DES enabled the extraction of a wide range of hydroxycinnamic acids and flavonol derivatives, whereas the choline-based DES was selective towards anthocyanins.

The higher extraction yields and selectivity that the extractions with DESs present can be explained by the damage that the DES provokes in the cell wall of the vegetable material. Scanning electron microscopy (SEM) was used to analyse the surface of the material before and after the extraction with DESs and compared with the extraction performed with conventional organic solvents, the materials extracted with DESs showed more pores and cracks due to the penetration of the DES into the cell structure due to the high hydrogen bonding, van der Waals forces, and ionic interactions [173,174]. Chen et al. [175] studied the mechanism of extraction of artemisinin from the leaves of *Artemisia annua* L. by SEM and ^1^H nuclear magnetic resonance and verified that the hydrogen reformation and the plant tissue destruction play vital roles during the extraction process. The penetration of the DESs in the cell wall due to strong interactions is also possible when hydrophobic DESs are used. In this case, the van der Waals interactions are the main mechanism [176]. The extraction performance is enhanced when the DESs are used in conjunction with UAE. In these cases, there is a synergetic effect between the cavitation phenomenon of the ultrasound and the ability of the DESs to bond to the target molecules [166,177,178].

The extracts with DESs also presented better results in terms of antimicrobial activity [165,174,179], antiproliferative activity in tumour Caco-2 cells and normal human keratinocyte cells [180], lower toxicity and increased bioavailability of the target compounds when administered to rats [181], higher antioxidant effects [179], and higher anti-inflammatory effects, as well as inhibitory effects against α-glucosidase and pancreatic lipase [182] when compared to the extracts from the traditional solvents. The enhanced biological activity of the DESs extracts can be explained by the higher content of bioactive compounds [182,183]. Duarte et al. [184] studied the effect of extracts rich in polyphenols from a UAE based DES extraction and concluded those extracts showed a large antioxidant activity and a significant effect on the growth of Gram-positive and Gram-negative bacteria. The biological activity of the DESs extracts opens the possibility of using these extracts in pharmaceutical industry, to prevent and treat diseases or to develop new drugs.

Another important advantage of the extracts containing DESs is their stability. Some studies were performed to study this parameter and found that the DESs extracts have a longer shelf-life when compared with conventional solvents extracts [129,180]. This stabilization was due to the ability of the DESs to stabilize the phenolic compounds [185]. Lee et al. [160] used leaves from kale to extract several health-promoting phytochemicals using natural DESs with different hydrophilicity/hydrophobicity. The DES extracts provided the greatest stability of the bioactive polyphenols by retaining 91.7% and 88.6% of the original content after 30 days of storage at 4 °C and 25 °C, respectively.

One of the main drawbacks in using the DESs in an industrial scale is the difficulty to remove the DESs from the extracts and to recycle them for further use; however, new methods are being developed to be able to separate the DESs from the extracts and to reuse them for subsequent extractions. Anstiss et al. [129] screened twenty-two hydrophobic DESs to extract fatty acids and, instead of removing the DES from the extracts, reused the entire extract five times without losing any extraction efficiency. Another approach was used by Lanjekar et al. [186], who used a macroporous resin to entrap the compound of interest, glycyrrhizin acid, and separate it from the extracts. They reused the DESs in two cycles with more than 90% extraction efficiency. Water as an anti-solvent was used to recover and recycle DES based-on choline chloride used to extract rutin form from *Saphora japonica* L. with a yield of 94.9%. The DESs were reused at least three times without loss of extraction yield [187]. The anti-solvent method was also used by Liu et al. [166] to recover 71.7% of scutellarin after the DES extraction. Biphasic systems were also developed to separate the DESs from the extracts. These systems comprised an aqueous DES phase and a second organic phase (e.g., ethyl acetate). Using this system, the target compounds are extracted to the aqueous phase and then transferred to the hydrophobic phase. In this way, the target compounds are separated from the DES, and at the same time, the extraction yield is increased [188]. Other approaches were developed to separate the DESs from the extracts based on temperature [189], pH [190], and CO_2_ [169]. Tang et al. [189] prepared hydrophobic temperature-switchable DESs to extract *Lycium barbarum* polysaccharides. After the extraction, the temperature was changed to separate the two phases, the aqueous, rich in polysaccharides and the hydrophobic one with the DESs. The authors reused the DESs five extraction cycles with a percentage of recovery after the fifth cycle of 80.2%. Ca et al. [190] used pH responsive polymeric DESs with a phosphate salt to extract aromatic amino acids. By adjusting the pH, the DESs were separated and reused for further extractions. Wang et al. [169] synthesized CO_2_-responsive DESs to extract hesperidin from orange peel. The reversible phase transformation of the DES solution was achieved by bubbling CO_2_/N_2_ in the DES solution to recover the hesperidin from the top phase.

The type of extraction method used is the most significant factor affecting the extraction outcome. Methods such as MAE and UAE are often used in conjunction with DESs. These methods drastically reduced the solvent requirement and extraction time, thereby minimizing energy consumption and cost compared with traditional extraction processes [191]. Additionally, the cavitation phenomenon of the UAE in junction with DESs proven to be highly effective on disrupting the cell wall, increasing the extraction efficiency [173].

MCAE using DESs was used to extract bioactive compounds from tea leaves, and the results showed that the extraction was complete within 20 s; therefore, this method has the potential to be a powerful tool for efficient extraction of thermally sensitive bioactive compounds [192].

The choice of the DESs to use in the extraction process is normally based on trial-and-error approaches or acquired knowledge. However, some theoretical approaches have been used to choose the DESs. These approaches assume that the best DES to extract a pre-determine compound is the one that facilitates a higher number of interactions (hydrogen bonds, van der Waals/electrostatic forces, etc.) with the target compound. Fan et al. [175] used COSMO-RS (short for Conductor-like Screening MOdel for Real Solvents) to design DESs capable of extract artemisinin from the leaves of *Artemisia annua* L. The program was used to predict the thermophysical properties and extraction performance of the DESs by simulating the intermolecular forces of the target in the mixed system. COSMO-RS was also used to screen twenty-two hydrophobic DESs by predicting their interactions with the two omega-3 polyunsaturated fatty acids [129]. In both cases, the DESs screen showed to be efficient and selective towards the target compound.

**Table 1:** Application of DESs as extraction solvents of bioactive compounds from natural sources in the year 2023.

Best DES(s) (Molar Ratio)	Natural Source	Target Compound	Extraction Technique(s)	Reference
ChCl:PG (1:2)	Wolfberry	Rhamnogalacturonan-I (RG-I) pectin	CE	[174]
LA:water	Vine shoots	Phytochemicals (proanthocyanins, stilbenes, hydroxycinnamic acids, and flavonols)	UAE/CE	[184]
ChChl:MA (1:1) ChChl:GLY (1:2)	Orange	Bioactive compounds and ascorbic acid	CE	[185]
LA:GLY (1:2)	*Evodia lepta*	Alkaloids	MAE	[188]
ChChl:EG (1:3)	Abalone viscera	Polysaccharides	UAE	[193]
TET: LAU (1:1)	*Lycium barbarum*	Polysaccharides	UAE	[189]
Terpenoid-based	*Rosmarinus officinalis* L.	Bioactive oxidants	UAE	[176]
CAM:GLY (1:1)	*Micromelum minutum*	Polysaccharides	CE	[194]
ChChl:GLY (1:2) ChCl:LAC (1:3) ChCl:CA (1:1).	Apple	Bioactive compounds	UAE	[164]
BetHCl:EG (1:10)	Kiwifruits	Antioxidants	CE	[162]
[N4444]Cl:AA (1:1).	---	Aromatic amino acids	---	[190]
TEA:4-MP (1:1)	Orange	Hesperidin	UAE	[169]
ChCl-ACA (1:2)	Peanut	Flavonoids	UAE	[178]
BET-LAC	Astragalus-Safflower	hydroxysafflor yellow A, anhydrosafflor yellow B, eleutheroside B, calycosin-7-O-glucoside, kaempferol-3-O-rutinoside, ononin, calycosin, astraganoside, carthamin	UAE	[181]
LACT: NAACE:H_2_O (3:1:2) ChCl:OA (1:1)	Blueberry	Phenolic compounds	UAE	[172]
ChCl:ACE (1:4)	Erigerontis Herb	Scutellarin	UAE	[166]
ChCl:CA (1:1)	*Curcuma longa*	Curcuminoids	MAE	[191]
BET:GLY (1:3)	Kale	Polyphenols	Solid/Liquid	[160]
BET:MA:PRO (1:1:1)	Propolis	Bioactive compounds	UAE	[195]
ChCl:Gly (1:2) ChCl:URE(1:2)	*Rhamnus alaternus*	Polyphenols	CE	[179]
ChCl:EG(1:2)	*Sophora japonica* L.	Rutin	CE	[187]
BENZAC:FEN (1:4)	*Artemisia annua* L.	Artemisinin	CE	[187]
β-ALA:MA:H_2_O (1:1:3)	Mango	Phenolic compounds	CE/ UAE	[171]
CA:MA:H_2_O (1:1:10)	Persimmon	Fibers/Antioxidants	UAE	[196]
ChCl:GLY (1:1)	Avocado	Phenolics/Carbohydrates	MAE	[197]
ChCl:GLY (1:2)	Kiwifruits	Polyphenols	UAE	[182]
ChCl:GLY (1:2)	Spice	Polyphenols	UAE	[198]
ChCl:ACA (1:4)	Tarragon	Bioactive compounds	CE	[199]
LAC:GLY (1:2)	Foxtail millet Husk	Bioactive compounds	UAE	[173]
ChCl:GLY (1:1)	Violet Potato	Bioactive compounds	UAE/MAE	[180]
BET:TEG (1:2) ChCl:PROP (1:2)	Coffee ground	Polyphenols	CE	[165]
BET: FRU (1:1)	Dark Chocolate	Bioactive compounds	UAE	[167]
ACA:GLU (2:1) ACA:GLY (2:1)	Tea	Tannins/Flavonoids/Terpenoids	UAE/EAE	[183]
MEN:LID (1:1)	*Perna* *canaliculus*	Omega-3	CE	[129]
DDBAC:LA (1:3)	Gardenia	Bioactive Compounds	CE	[200]
[N_1 1 16 (2OH)_^+^][Br^−^]:THY (1:2)	*Cannabis sativa* L.	Phytochemicals	Microextraction	[161]
ChCl:BUT (1:4)	*C. vulgaris*	Bioactive compounds	CE	[168]
ChCl:MA (1:1) ChCl:LAC (1:3)	*Aralia elata*	Triterpene Saponins	CE	[201]
CA:GLY:H_2_O (1:4:10/15/20)	*Chamaenerion angustifolium* (L.) Scop.	Bioactive compounds	UAE	[202]
CA:MAL (1:2)	Nettle	Bioactive Compounds	UAE	[203]
ChCl:LAC (1:1)	*Iris sibirica* L.	Bioactive compounds	CE	[204]
ChCl:LAC (1:2)	Edible Feijoa	Flavonoids	CE	[205]
ChCl:LAC (1:4)	Mexican Oregano	Flavonoids	UAE	[206]
ChCl-PHE (1:3)	Hop	Polyphenols	CE/UAE/UHE	[207]
ChCl:GLU (1:0.8)	*Capsicum chinense*	Polyphenols	UAE	[170]
ChCl:LAC (1:1)	*Glycyrrhiza glabra*	Glycyrrhizic acid	MAE	[170]
ChCl:MA (1:2) ChCl:CA (1:2) ChCl:4BUT (1:2)	Fenugreek	Flavonoids	UAE	[208]
ChCl:LAC (1:2)	black mulberry	Flavonoids/Phenolics	UAE	[177]
ChCl:4BUT (1:2)	Tea	Flavonoids/Alkaloids/Catechins	MCAE	[192]
ChCl:LAC (3:1)	Phaeophyceae	Phlorotannins	UAE	[209]

## 5. Conclusions and Future Perspectives

The search for more sustainable extraction techniques has been subject of intense research in the past decades. The extraction techniques evolved and nowadays the scientific and industrial community has at their disposal extraction techniques more effective and environmentally friendly. Techniques such as MAE and UAE become increasingly popular as extraction techniques due to their high yields and low solvent and energy consumption. However, part of these techniques still uses organic toxic solvents.

Neoteric solvents have emerged as possible substitutes for organic solvents. They are green solvents due to their low toxicity and high biodegradability. Within the neoteric solvents, DESs gain special attention due to their low cost, readily available materials, and easy production. From their properties, their high solubility capability for hydrophobic and hydrophilic compounds may be one of the most important for their use as extraction solvents. Moreover, the availability of a high number of possible HBA and HBD combinations, modulating their properties as solvents, make DESs very attractive to target specific bioactive compounds. Is has been shown in several works that the use of DESs with extraction techniques, such as UAE, increases the extraction efficiency due to the capability of the DESs to form hydrogen bonds with the bioactive compounds. Due to their low toxicity, DESs can be used in conjunction with the bioactive compounds in pharmaceutical or cosmetic products; nevertheless, there are already some studies in which the DES was separated from the extract allowing it to be reused several times without losing its ability to extract the target compound. DESs have also shown a high ability to stabilize bioactive compounds, which is a huge advantage since many bioactive compounds are known to have low stability in most of the solvents, including water.

Despite the work developed over the last decade on understanding DESs, there are still some areas that need to be deepened. The toxicity and/or biodegradability of the DESs are still far from being fully understood in terms of what determines the synergetic effects of the DESs upon their use. The structure–activity relationship of the DESs needs to be explored to establish a more comprehensive way to choose the right DES for a certain application. Regarding the use of the DESs as extraction solvents, more studies are needed with different extraction techniques. For example, the use of DESs in SWE is still almost unexplored. It is also necessary to deepen the knowledge about the possible synergistic effects that may occur between the DES and the bioactive compounds, whether they affect the bioavailability of the bioactive compounds in the extract, the way that they impact the stability of the extract, and their toxicity in the final extract.

Overall, DESs have been proven to be good solvents for the extraction of bioactive compounds, regardless of the natural matrix or the extraction technique used, and should be perused as alternatives to toxic organic solvents.

## Data Availability

Data sharing is not applicable.

## Abbreviations

BUT	1,2-Butanodiol
PROP	1,2-Propanediol
4BUT	1,4-Butanodiol
4-MP	4-Methoxyphenol
ACE	Acetamide
ACA	Acetic Acid
AA	Acrylic Acid
API	Active Pharmaceutical Ingredient
BENZAC	Benzoic acid
BET	Betaine
BetHCl	Betaine hydrochloride
[N1 1 16 (2OH)+][Br−]	Choline Bromide Salt
ChC l	Choline chloride
CA	Citric Acid
CAM	Citric Acid Monohydrate
COSMOS-RS	Conductor-like Screening Model for Real Solvents
CE	Conventional Extraction
DES	Deep Eutectic Solvent
DESs	Deep Eutectic Solvents
GLU	D-Glucose
DDBAC	dodecyldimethylbenzylammonium chloride
EAE	Enzymatic-Assisted Extraction
EG	Ethylene glycol
FEN	Fenchyl alcohol
FAE	Fermentation-Assisted Extraction
FA	Formic acid
FRU	Frutose
Tg	Glass Transition Temperature
GLY	Glycerol
HBA	Hydrogen Bond Acceptor
HBAs	Hydrogen Bond Acceptors
HBD	Hydrogen Bond Donor
HBDs	Hydrogen Bond Donors
IUPAC	International Union of Pure and Applied Chemistry
Ils	Ionic Liquids
LACT	Lactate
LAC	Lactic Acid
LAU	Lauric acid
LA	Levulinic acid
LID	Lidocaine
MA	Malic acid
MAL	Maltose
MCAE	Mechanochemical Extraction
MEN	Menthol
MAE	Microwave-assisted extraction
NADESs	Natural Deep Eutectic Solvents
OA	Oxalic acid
PHE	Phenol
PDESs	Polymeric Deep Eutectic Solvents
PQDESs	Poly-Quasi Deep Eutectic Solvents
PRO	Proline
PG	Propylene Glycol
NAACE	Sodium Acetate
SWE	Subcritical Water Extraction
SFE	Supercritical Fluid Extraction
[N4444]Cl	Tetrabutylammonium chloride
TET	Tetracaíne
THEDESs	Therapeutic Deep Eutectic Solvents
THY	Thymol
TEA	Triethanolamine
TEG	Triethylene glycol
UHE	Ultrasonic Homogenizer Extraction
URE	Urea
UAE	Ultrasound liquid extraction
β-ALA	β-Alanine
